# Electrical Resistivity and Microwave Properties of Carbon Fiber Felt Composites

**DOI:** 10.3390/ma15238654

**Published:** 2022-12-05

**Authors:** Marina Tretjak, Sandra Pralgauskaitė, Jonas Matukas, Artyom Plyushch, Jan Macutkevič, Jūras Banys, Blagoj Karakashov, Vanessa Fierro, Alain Celzard

**Affiliations:** 1 Physics Faculty, Vilnius University, Sauletekio av. 9, LT-10222 Vilnius, Lithuania; 2 French National Centre for Scientific Research, Université de Lorraine, CNRS, IJL, 88000 Épinal, France

**Keywords:** carbon fibers, electrical resistivity, electromagnetic shielding

## Abstract

We present studies on the microwave properties, electrical resistivity, and low-frequency (10 Hz–20 kHz) noise characteristics in the temperature range of 78 K to 380 K of composite materials made from bisphenol A-based epoxy resin and carbon fiber felts. Two types of carbon fibers were used, derived from polyacrylonitrile or regenerated cellulose. We show that these structures are suitable for electromagnetic shielding applications, especially in the direction parallel to the carbon fibers. The low-frequency voltage fluctuations observed in these materials are of the 1/*f^α^*, and the noise intensity is proportional to the square of the voltage. The characteristics of the investigated materials show an instability in the temperature range from 307 K to 332 K. This effect is followed by an increase in resistivity and noise intensity, but it does not change the character of the noise, and this instability vanishes after a few repeated heating and cooling cycles.

## 1. Introduction

Nowadays, microwaves are widely used in various fields of human life, including telecommunications and electronics [1]. Electromagnetic interference becomes a crucial problem because some devices operate in the same or neighboring frequency ranges. In addition, the power of microwave radiation increases with the number of devices operating in the same space and by the rapid advancement of telecommunications, where higher frequencies and thus greater powers are required to transmit a large amount of information. Carbon felts (nonwovens based on carbon fibers) are widely used in various applications due to their unique structural and physical properties, including low electrical resistivity, good mechanical strength, and excellent chemical resistance, as well as commercial availability, lightness, and ease of handling and processing, which allows these structures to be used for various electromagnetic shielding applications [2,3,4,5].

Composites with epoxy resin as the polymer matrix and carbon particles as the filler are used in a variety of modern technologies, such as functional layers in electronic devices; as strong electrostatic protective and charge-elimination materials in the aerospace industry, robotic structures and automotive components; and in the rapidly expanding lithium-ion battery industry [6,7,8,9,10,11]. Such materials indeed have special properties of resistance to mechanical stress, humidity and aggressive environments, radiation, high temperature, and thermal cycling [12,13,14]. Carbon fiber-reinforced polymer composites have very high specific strength and modulus of elasticity but are subject to mechanical damage due to heat; the materials undergo mechanical stress as the temperature increases, resulting in delamination between the layers [13]. This leads to shrinkage of the carbon fibers, so that the linear dimensions in the direction of reinforcement decrease [11]. Commercial applications of new materials require stability of their characteristics and a complete understanding of the physical phenomena that take place under varying environmental and operating conditions. Electrical conduction in such composite materials based on a dielectric matrix and a conductive filler can be achieved in two ways via charge carrier transport: within the conductive structure of the filler or through the dielectric matrix by tunneling between the filler particles [15,16]. The characteristics of low-frequency electrical noise are well known to be extremely sensitive to the physical processes corresponding to charge carrier transport mechanisms in various materials and devices [17,18,19,20]. However, papers on low-frequency electrical fluctuations in carbon composite devices are very rare. There are also no papers devoted to noise analysis in carbon felt composites.

In this paper, we present a study of the microwave properties, electrical resistivity, and low-frequency noise characteristics of carbon fiber felt and epoxy resin composites with the aim of clarifying the changes in electrical conductivity mechanisms in these materials as a function of temperature, and explaining the possibility of using these composites for electromagnetic shielding applications.

## 2. Materials and Methods

### 2.1. Carbon Fiber Felt Composites

Composite materials based on carbon fiber felt (CFF) and epoxy resin were prepared as follows. Two types of carbon fibers were used, obtained from different precursors. On the one hand, fibers derived from regenerated cellulose (RC) were kindly provided by Beijing Great Wall (China) under the trade name RSF2. They were obtained by heat treatment between 2200 and 2400 °C under an inert atmosphere and have a carbon content equal to or higher than 99%, and an ash content equal to or lower than 0.5%. On the other hand, fibers derived from polyacrylonitrile (PAN) were kindly provided by CeraMaterials. They were obtained by heat treatment at 2000 °C and have the same chemical composition as the previously described ones. These fibers also differed in diameter, at 10.70 ± 2.36 µm for the RC-derived fibers and 19.71 ± 1.49 µm for the PAN-derived fibers. More information about these fibers can be found elsewhere [21,22].

**Figure 1:**
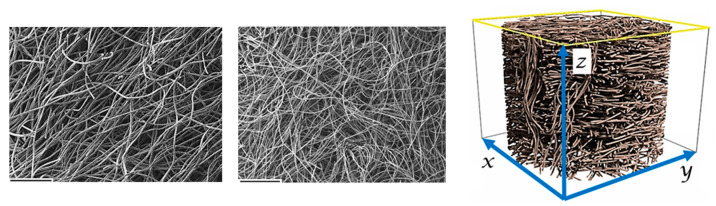
“In-plane” SEM micrographs of PAN and RC carbon felts, and schematic representation of soft felt samples, with *x*, *y*, *z* orientations of the fibers: *x*, *y* orientations are defined as “in-plane”; *z* orientation is defined as “out-of-plane”. The scale bar in the lower left corner of the SEM images is 500 µm long.

The carbon fibers were laid/dispersed into layers and then held together by needle punching. Soft and anisotropic graphite carbon felts were thus obtained. In-plane SEM views of the felts of both types of fibers are presented in Figure 1. The felts were then dipped in a bisphenol A epoxy resin, used as a binder, and cured. Samples for measurement were cut along the *x*, *y*, and *z* axes. The *x* and *y* directions correspond to the “in-plane” direction, i.e., the bedding (horizontal) plane of the fibers, in which there is no preferential orientation. The *z* axis is the orthogonal direction defined as “out-of-plane” (Figure 1). Two ways of attaching the contacts to the samples were used: in the *xy* plane (samples designated “PAN II” and “RC II”), and in the *xz* plane (“PAN X” and “RC X”). In total, four types of samples were prepared.

The mechanical and thermal properties of these felts have been detailed elsewhere [2]. Briefly, the elastic moduli of the ex-RC and ex-PAN fiber felts are 0.142 and 0.187 MPa, respectively, with total porosities of 94.6% and 93.8%, respectively. Their average thermal conductivities at room temperature are 0.143 and 0.806 W m^−1^ K^−1^ in the in-plane direction, respectively, and 0.041 and 0.026 W m^−1^ K^−1^ in the out-of-plane direction, respectively.

### 2.2. Measurement Techniques

Microwave studies were performed in the 26–35 GHz frequency range with a R2-408R scalar network analyzer. The complex dielectric permittivity was determined by the thin cylindrical rod method, measuring the reflection and transmission moduli [23]. The electrical resistance of the samples was measured by a Keysight Technologies B1500A semiconductor analyzer (Santa Rosa, CA, USA). Voltage fluctuations were measured by a low-noise measurement system consisting of a low-noise amplifier a filter system, and an analog-to-digital converter (NI TM PCI 6115, provided from National Instruments (Austin, TX, USA). The noise voltage spectral density was evaluated by comparison with the thermal noise of a reference resistor:

(1) $$S_{U}=\frac{\overset{´}{U^{2}}-\overset{´}{U_{s}^{2}}}{\overset{´}{U_{ref}^{2}}-\overset{´}{U_{s}^{2}}}4k_{B}TR_{ref},$$

where $\overset{´}{U^{2}},\overset{´}{U_{ref}^{2}}$, and $\overset{´}{U_{s}^{2}}$ are the dispersions of the voltage fluctuations of the sample under study, of the reference resistor (both including the noise of the measurement system), and of the measurement system in the narrow frequency band, respectively. *T* and *R_ref_* are the absolute temperature and resistance of the reference resistor, respectively, and *k_B_* is the Boltzmann constant. The constant current mode was guaranteed by choosing a load resistance at least 100 times the resistance of the sample under study. The noise characteristics were investigated over a frequency range from 10 Hz to 20 kHz and at temperatures from 78 K to 380 K. The measurements of the low-frequency voltage fluctuations were carried out in a specially shielded laboratory (Faraday cage) to avoid interference from external electromagnetic disturbances. 

## 3. Results

### 3.1. Electrical Transport

**Figure 2:**
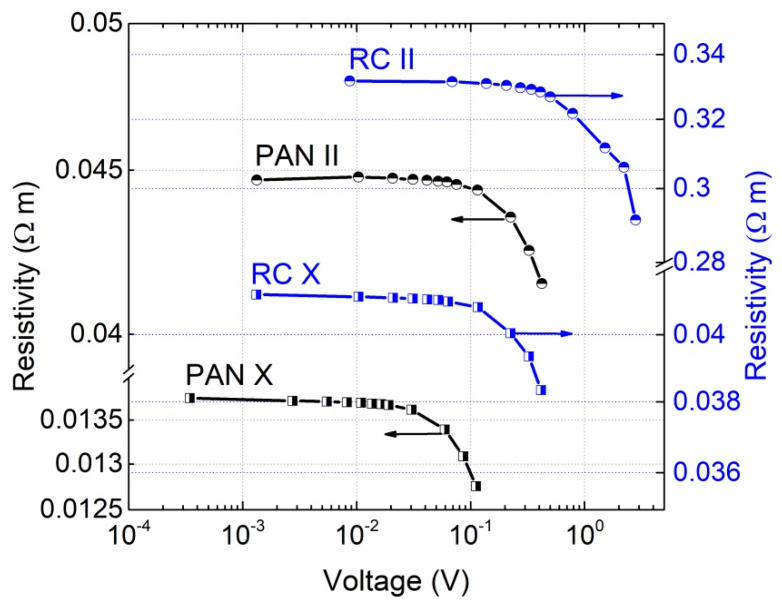
Resistivity dependence of the investigated composites with respect to room temperature voltage.

The resistance characteristics of the investigated CFF composites are typical of materials based on a dielectric matrix and conductive particles above the percolation threshold, with the resistance being constant at low voltages and starting to decrease at higher voltages (Figure 2). Electrical conduction in such composites is based on two processes: charge carrier transport in the carbon fibers and tunneling between the fibers. The probability that the carrier will tunnel through the barrier formed by the dielectric matrix increases strongly at a particular voltage [24,25]. For the materials studied, this voltage varies from 0.1 V to 1 V. 

**Figure 3:**
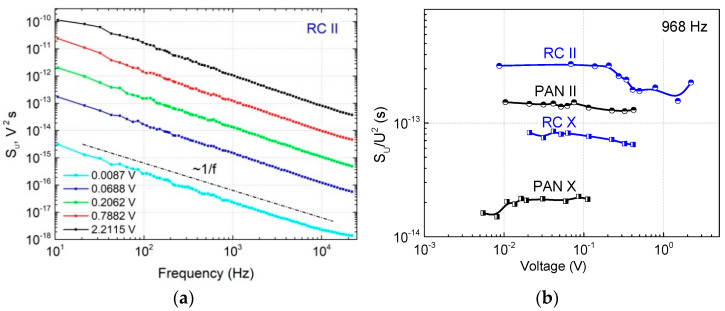
Voltage noise spectra for the RC II composite at different voltages (**a**) and voltage noise spectral density versus voltage dependencies for different samples (**b**) at room temperature.

The low-frequency voltage noise spectra of the investigated composites are of the same $1/f^{\alpha }$ type over the voltage and temperature ranges studied. The spectra for the RC II sample are presented in Figure 3 as an example. The nature of the $1/f^{\alpha }$ type noise is explained by the superposition of many processes with widely distributed characteristic times that randomly change the number of free charge carriers. The voltage noise intensity (noise spectral density) is proportional to the square of the voltage (Figure 3), indicating resistance fluctuations. Therefore, the dominant mechanism of charge carrier transfer at room temperature in the investigated composites is a process of charge carrier generation and recombination through the traps formed by defects in the carbon fibers.

**Figure 4:**
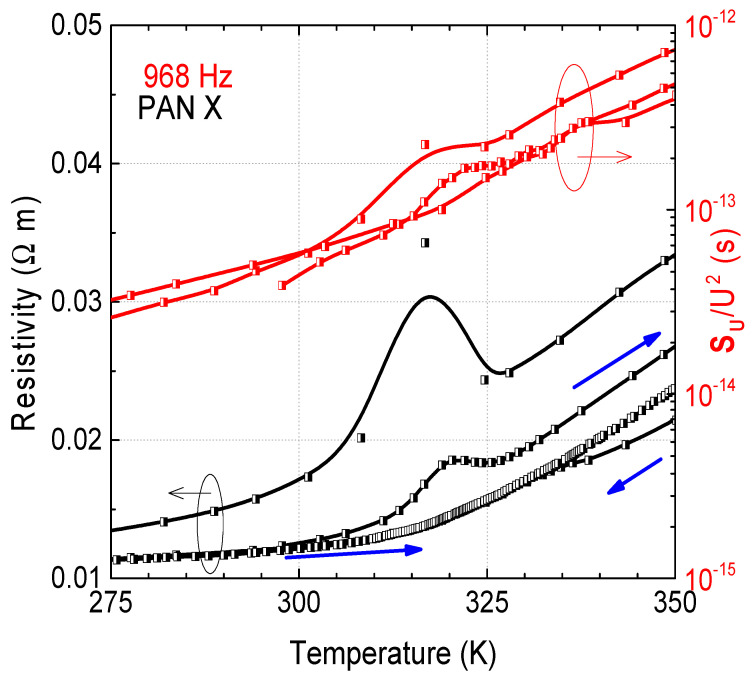
Dependencies of resistivity and voltage noise spectral density at 968 Hz of the PAN X sample on temperature at 31 mV (blue arrows indicate the directions of temperature changes).

The resistivity and low-frequency noise characteristics vary weakly with temperature in the low-temperature range. Both resistivity and noise intensity begin to increase more significantly at temperatures above approximately 250 K. This phenomenon is usually observed in epoxy resin composites with conductive particles and is related to the rapid matrix expansion, the thermal expansion coefficient of which is about 10^−4^ K^−1^ at temperatures above 300 K [23]. Since the thermal expansion coefficient of carbon fibers, which is highly anisotropic, is very different from that of the resin, at about 10^−6^ K in the axial direction and about 10 times more in the perpendicular direction [23], the increase in temperature leads to a partial destruction of the carbon fiber network. The more interesting temperature range is between 307 K and 332 K, where a peak in resistivity and noise intensity is observed (Figure 4). This peak is more noticeable in the PAN samples, while just a small step in resistivity and noise spectral density is observed for the RC samples. This could be explained by the fact that ex-PAN fibers are more graphitized than ex-RC fibers [22], and that their thermal expansion coefficients are different in the direction parallel and perpendicular to the fiber axis. In other words, their anisotropic character is expected to be more pronounced.

It was also observed that, after repeated heating-cooling measurement cycles, this peak decreases and almost disappears. We suppose that the reason for this instability is the reduction in the linear dimension of the carbon fibers in the direction of the composite reinforcement (when the matrix expands), which leads to the increase in resistivity. The carbon fibers lose their ability to shrink after a few repeated temperature cycles [11]. The intensity of this effect depends on the nature of the fiber material or its diameter; it is larger for composites with polyacrylonitrile-based carbon fibers that are almost twice as thick as those derived from regenerated cellulose, and weakly depends on the direction of current flow. This can also be explained by the fact that, as said above, the anisotropy of the properties is more pronounced on ex-PAN fibers, which are fully graphitized, in contrast to ex-RC fibers, while nominally called graphitized because of the high-temperature treatment they have undergone, in reality they are not [22]. The noise intensity has the same temperature dependence as the resistivity, and the voltage noise spectra retain the same $1/f^{\alpha }$ type in this temperature region, showing that no new noise origin arises due to matrix expansion and fiber shrinkage.

### 3.2. Microwave Properties

**Figure 5:**
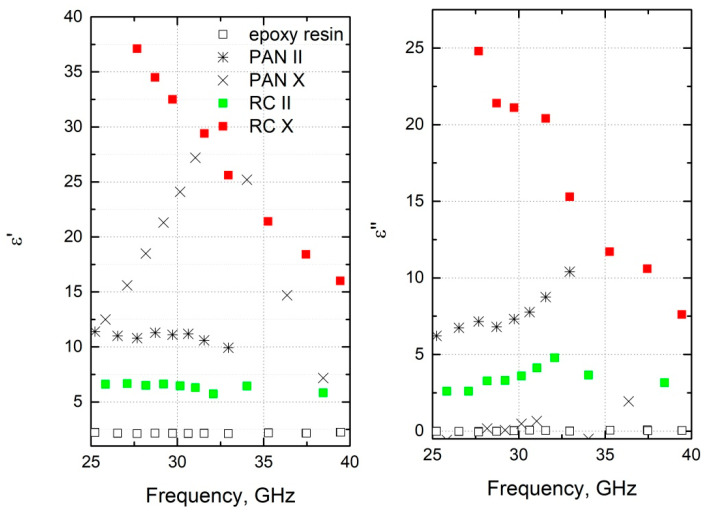
Microwave spectra of carbon fiber felt composites.

**Figure 6:**
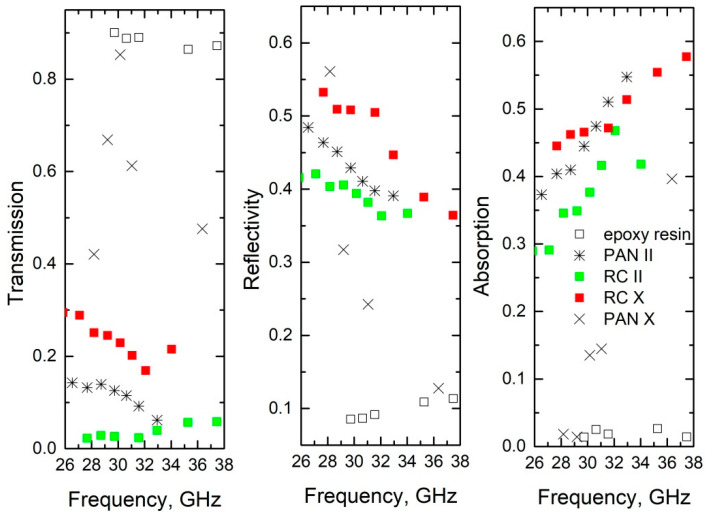
Reflection, transmission, and absorption of carbon fiber felt composites.

The microwave spectra of the carbon fiber felt composites are presented in Figure 5. For the composites with RC inclusions in the perpendicular direction, the values of the real and imaginary parts of the dielectric permittivity were quite high (ε′ = 30 and ε″ = 21 at 30 GHz). Therefore, these structures can be suitable for electromagnetic shielding applications [24,25,26,27]. Indeed, if we consider a thin planar layer in free space with perpendicular incident electromagnetic irradiation, then the scattering parameters can be calculated according to Equations (2) and (3):

(2) S11=−jkzk2z2−1sink2zτ2jkzk2zcosk2zτ+kzk2z2+1sink2zτ,

(3) S21=2kzk2z2k2zkzcosk2zτ+jkzk2z2+1sink2zτ,

where *k_z_* = 2π/λ and *k*_2*z*_ = 2πε^0.5^/λ are the wave numbers in vacuum and in the sample, respectively, and τ is the thickness of the composite layer. The absorption of the layer was calculated as *A* = 1 − (*S*_11_)^2^ − (*S*_21_)^2^. The results of the calculations obtained for the composite layers with a thickness of 1 mm are summarized in Figure 6.

It can be concluded that the absorption of some of these composite structures is quite high, for example, that of the RC X sample at 35 GHz is 55%. It is comparable to the best absorption of ceramics with SiC or Ag nanoinclusions, or hybrid carbon magnetic/carbon nanostructures [26,28,29,30,31,32]. Measurements in different directions reveal strong anisotropy of the electromagnetic response. In particular, the transmission for the PAN sample decreases from 70% down to only 17%, while for RC sample the decrease is from 25% down to almost 0. That gives such structures prospect for applications as polarizers in the microwave frequency range. In comparison with the carbon nanotube-based counterparts [33,34], the proposed structures provide higher degrees of anisotropy in the electromagnetic response. However, nanotubes are more suitable for the THz range [35,36,37]. This, in combination with an easier preparation procedure, makes the proposed materials a perfect alternative to nanotube-based composites for the microwave frequency range.

The studied materials can be used for shielding applications both with and without backreflectors. Moreover, by applying the Salisbury screen λ/4 method and backreflectors [26,38] to the present composites, even 100% absorption can be achieved.

## 4. Conclusions

A study of the microwave properties in terms of electrical resistivity and low-frequency noise characteristics has been performed on composites based on epoxy resin and carbon fiber felts.

The low-frequency voltage fluctuations of these materials are of 1/*f^α^* type, and the origin of these fluctuations is the fluctuation of resistance due to random generation and recombination of charge carriers in the carbon fiber structure.

Expansion of the matrix and shrinkage of the carbon fibers were observed as the temperature was varied between 307 K and 332 K. This effect was followed by an increase in resistivity and noise intensity, but it did not change the character of the noise or the dominant conduction mechanism.

These composite materials based on epoxy and carbon felts have been shown to be suitable for electromagnetic shielding applications.

## Data Availability

Not applicable.

## References

[1] Ryu S.H., Kim H., Park S.W., Kwon S.J., Kim S., Lim H.R., Park B., Lee S.B., Choa Y.H. (2022). Millimeter-scale percolated polyethylene/graphene composites for 5G electromagnetic shielding br. ACS Appl. Nano Mater..

[2] Karakashov B., Taghite M.B., Kouitat R., Fierro V., Celzard A. (2021). Mechanical and thermal behavior of fibrous carbon materials. Materials.

[3] Yin Z.T., Cai W., Lu J.Y., Yu B., Wang B.B., Song L., Hu Y. (2022). Cost-effective graphite felt and phosphorous flame retardant with extremely high electromagnetic shielding. Compos. Part B-Eng..

[4] Guo Z.Z., Ren P.G., Zhang Z.P., Dai Z., Hui K.D., Yan H.H. (2021). Simultaneous realization of highly efficient electromagnetic shielding and human motion detection in carbon fiber felt decorated with silver nanowires and thermoplastic polyurethane. J. Mater. Chem. C.

[5] Zhou M., Wang J.W., Wang G.H., Zhao Y., Tang J.M., Pan J.X., Ji G.B. (2022). Lotus leaf-inspired and multifunctional Janus carbon felt@Ag composites enabled by in situ asymmetric modification for electromagnetic protection and low-voltage joule heating. Compos. Part B-Eng..

[6] Faulstich de Paiva J.M., Mayer S., Rezende M.C. (2005). Evaluation of mechanical properties of four different carbon/epoxy composites used in aeronautical field. Mater. Res..

[7] Han F.D., Yao B., Bai Y.J. (2011). Preparation of Carbon Nano-Onions and Their Application as Anode Materials for Rechargeable Lithium-Ion Batteries. Phys. Chem..

[8] Aref A.R., Chen S.W., Rajagopalan R., Randall C. (2019). Bimodal porous carbon cathode and prelithiated coalesced carbon onion anode for ultrahigh power energy efficient lithium ion capacitors. Carbon.

[9] Yoon N., Lee Y., Kang D., Min J., Won J., Kim M., Kan Y.S., Kim S.-H., Kim J.-J. (2011). Modification of hidrogenated bisphenol A epoxy adhesives using nanomaterials. Int. J. Adhes. Adhes..

[10] Baker A., Dutton S., Kelly D. (2004). Composite Materials for Aircraft Structures.

[11] Starcev O.V., Hristoforov D.A., Kliusnicenko A.B., Rumiancev A.F., Guniaev G.M., Raskutin A.E. (2002). Relaxation of temperature deformations of carbon fibers. Acad. Sci. Rep..

[12] Starcev V.O., Raskutin A.E., Starcev O.V., Guniaev G.M. (2009). Thermal Expansion of Carbon Fibers and Carbon Plastics Based on Them, Technique and Technology for the Production of Thermal Insulation Materials from Mineral Raw Materials.

[13] Choi I., Lee D.G. (2013). Surface modification of carbon fiber/epoxy composites with randomly oriented aramid fiber felt for adhesion strength enhancement. Compos. Part A Appl. Sci. Manuf..

[14] Luo R., Liu T., Li J., Zhang H., Chen Z., Tian G. (2004). Thermophysical properties of carbon/carbon composites and physical mechanism of thermal expansion and thermal conductivity. Carbon.

[15] Jin F.L., Park S.J. (2013). Recent Advances in Carbon-Nanotube-Based Epoxy Composites. Carbon Lett..

[16] Guadagno L., Raimondo M., Vittoria V., Vertuccio L., Lafdi K., De Vivo B., Lamberti P., Spinelli G., Tucci V. (2013). The role of carbon nanofiber defects on the electrical and mechanical properties of CNF-based resins. Nanotechnology.

[17] Palenskis V., Matukas J., Vyšniauskas J., Pralgauskaitė S., Shtrikman H., Seliuta D., Kašalynas I., Valušis G. (2013). Analysis of noise characteristics of GaAs tunnel diodes. Fluct. Noise Lett..

[18] Palenskis V., Matukas J., Pralgauskaitė S., Seliuta D., Kašalynas I., Subačius L., Valušis G., Khanna S.P., Linfield E.H. (2013). Low-frequency noise properties of beryllium δ-doped GaAs/AlAs quantum wells near the Mott transition. J. Appl. Phys..

[19] Pralgauskaitė S., Palenskis V., Matukas J., Šaulys B., Kornijčuk V., Verdingovas V. (2013). Analysis of mode-hopping effect in Fabry-Pérot multiple-quantum well laser diodes via low frequency noise investigation. Solide-State Electron..

[20] Jones B.K. (1994). Low-frequency noise spectroscopy. IEEE Trans. Electron. Dev..

[21] Karakashov B., Toutain J., Achchaq F., Legros P., Fierro V., Celzard A. (2019). Permeability of fibrous carbon materials. J. Mater. Sci..

[22] Karakashov B., Fierro V., Mathieu S., Gadonneix P., Medjahdi G., Celzard A. (2019). Structural characterisation and chemical stability of commercial fibrous carbons in molten lithium salts. Materials.

[23] Zhao J.X., Ding K.H., Wei J.X. (1987). Research on thermophysical properties and anti-thermal-vibration properties of C/C composites. Acta Mater. Compos. Sin..

[24] Grigas J. (2009). Microwave dielectric spectroscopy of ferroelectrics. Ferroelectrics.

[25] Zeng X.C., Bergman D.J., Hui P.M., Stroud D. (1988). Effective-medium theory for weakly nonlinear composites. Phys. Rev..

[26] Gefen Y., Shih W.-H., Laibowitz R.B., Viggiano J.M. (1986). Nonlinear Behavior near the Percolation Metal-Insulator Transition. Phys. Rev. Lett..

[27] Plyushch A., Macutkevic J., Svirskas S., Banys J., Plausinaitiene V., Bychanok D., Maksimenko S.A., Selskis A., Sokal A., Lapko K.N. (2019). Silicon carbide/phosphate ceramics composite for electromagnetic shielding applications whiskers vs. particles. Appl. Phys. Lett..

[28] Chen Z., Zhang Y., Wang Z., Wu Y., Zhao Y., Liu L., Ji G. (2023). Bioinspired moth-eye multi-mechanism composite ultra-wideband microwave absorber based on the graphite powder. Carbon.

[29] Plyushch A., Maciulis N., Sokal A., Grigalaitis R., Macutkevic J., Kudlash A., Apanasevich N., Lapko K., Selskis A., Maksimenko S.A. (2021). 0.7Pb(Mg_1/3_Nb_2/3_)O_3_-0.3PbTiO_3_ phosphate composites: Dielectric and ferroelectric properties. Materials.

[30] Plyushch A., Macutkevic J., Kuzhir P., Sokal A., Lapko K., Selskis A., Banys J. (2019). Synergy effects in electromagnetic properties of phosphate ceramics with silicon carbide whiskers and carbon nanotubes. Appl. Sci..

[31] Palaimiene E., Macutkevic J., Banys J., Selskis A., Apanasevich N., Kudlash A., Sokal A., Lapko K. (2022). Phosphate ceramics with silver nanoparticles for electromagnetic shielding applications. Materials.

[32] Peng Q., Ma M., Chen S., Shi Y., He H., Wang X. (2023). Magnetic-conductive bi-gradient structure design of CP/PGFF/Fe_3_O_4_ composites for highly absorbed EMI shielding and balanced mechanical strength. J. Mater. Sci. Technol..

[33] Bychanok D.S., Kaqnygin M.A., Okotrub A.V., Shuba M.V., Paddubskaya A.G., Pliushch A.O., Kuzhir P.P., Maksimenko S.A. (2011). Anisotropy of the electromagnetic properties of polymer composites based on multiwall carbon nanotubes in the gigahertz frequency range. Jetp Lett..

[34] Sedelnikova O.V., Kanygin M.A., Korovin EYu Bulusheva L.G., Suslyaev V.I., Okotrub A.V. (2014). Effect of fabrication method on the structure and electromagnetic response of carbon nanotube/polystyrene composites in low-frequency and Ka bands. Compos. Sci. Technol..

[35] Zubair A., Tsentalovich D.E., Young C.C., Heimbeck M.S., Everit H.O., Pasquali M., Kono J. (2016). Carbon nanotube fiber terahertz polarizer. Appl. Phys. Lett..

[36] Bychanok D.S., Shuba M.V., Kuzhir P.P., Maksimenko S.A., Kubarev V.V., Kanygin M.A., Sedelnikova O.V., Bulusheva L.G., Okotrub A.V. (2013). Anisotropic electromagnetic properties of polymer composites containing oriented multiwall carbon nanotubes in respect to terahertz polarizer applications. J. Appl. Phys..

[37] Bârsan O.A., He G., Alkorre H., Stiens J., With G. (2017). Identifying anisotropy in seemingly random CNT networks using terahertz techniques. Compos. Sci. Technol..

[38] Tennant A., Chambers B. (2004). A single-layer tuneable microwave absorber using an active FSS, IEEE microwave and absorber using an active. IEEE Microw. Wirel. Compon. Lett..

